# Mechanical and Electrical Performance of Flexible Polymer Film Designed for a Textile Electrically-Conductive Path

**DOI:** 10.3390/ma14092169

**Published:** 2021-04-23

**Authors:** Agnieszka Tabaczyńska, Anna Dąbrowska, Marcin Masłowski, Anna Strąkowska

**Affiliations:** 1 OPTEX S.A., Oskara Kolberga 2, 26-300 Opoczno, Poland; 2 Department of Personal Protective Equipment, Central Institute for Labour Protection—National Research Institute, Wierzbowa 48, 90-133 Lodz, Poland; 3 Institute of Polymer and Dye Technology, Lodz University of Technology, Stefanowskiego 12/16, 90-924 Lodz, Poland; marcin.maslowski@p.lodz.pl (M.M.); anna.strakowska@p.lodz.pl (A.S.)

**Keywords:** smart textiles, electrically-conductive paths, polymer film

## Abstract

Electro-conductive paths that are mechanically resistant and stable during simulated aging cycles are promising, in relation to the non-invasive application in e-textiles in our everyday surroundings. In the paper, an analysis of the influence of electro-conductive filler, as well as ionic liquid on surface resistance is provided. Authors proved that depending on the tested variant, obtained surface resistance may vary from 50 kΩ (when 50 phr of Ag and [bmim][PF_6_] ionic liquid applied) to 26 GΩ (when 25 phr of Ag and [bmim][PF_6_] ionic liquid applied). The samples were also evaluated after simulated aging cycles and the stability of electric properties was confirmed. Moreover, it was proved that the addition of ionic liquids reduced the resistance of vulcanizates, while no significant influence of the extrusion process on conductivity was observed.

## 1. Introduction

Nowadays, integration of electronics with textile structures is of the highest interest in order to provide solutions that make smart our both everyday, as well as professionally used clothing (e.g., protective clothing). Such smart textiles and clothing are also called active, interactive and adaptive, as well as e-textiles [1,2,3]. They require the application of electro-conductive material designed to carry electrical signals and provide textile material with electrical or electronic functions, including sensory functions, regulation, signaling [2,4,5]. This research area is also called textronics, which essence is the integration of sensors, energy, electronics, signal processing and communication within the textiles and clothing, in order to give it additional functions. Textronics uses knowledge areas such as chemistry, textile, electronics, computer science and automation [5,6] with a focus on the introduction of the electrically-conductive elements into the fibrous structures in such a way that the final product (i.e., with electronic properties) does not differ visually from the standard product (i.e., without electronic properties) [3,7]. Textronic items integrated with the clothing can be used to monitor physical parameters and vital signs, such as undergarment temperature, heart rate, respiratory rate without compromising the comfort of use and performance of the organism [7,8,9,10,11,12].

According to the recent forecast, the smart clothing market is supposed to grow significantly with about 50% CAGR (compound annual growth rate) till 2024 [13]. This boom is observed mainly thanks to innovative processing technologies that enable to manufacture of washable, flexible, lightweight and robust e-textiles [14], as well as increasing demand for sensorized garments for continuous detection of physiological parameters [15]. 

Conventional textile materials are usually non-conductive, therefore, in order to provide fabrics with electrical conductivity, several techniques have been already used [14,16,17,18,19]. Several integration techniques of integration of electrically-conductive material with textile substrate have been also considered, such as embroidering, knitting, weaving, spinning, braiding, coating or printing [14]. Metallic (copper) electro-conductive transmission lines obtained on spun-bonded type polypropylene nonwoven by means of magnetron sputtering were proposed by Nowak et al. [16]. The authors indicated that such lines demonstrate surface resistivity at the level of 0.2 Ω. Tokarska [12] analyzed both woven and knitted fabrics and discovered that samples with a smooth surface allow us to obtain higher electric conductivity than those with a rough one. Another approach was proposed by Stempień et al. [18] who used the inkjet-printing technique to deposit silver nanoparticle ink on various textile fabric surfaces. Authors proved that this method is able to ensure very low surface resistance which additionally performs good resistance to bending, washing and dry-cleaning cycles. Inkjet printing of reactive silver ink on textiles was also an area of interest of Shahariar et al. [20]. Authors highlighted challenges that they had to face due to the porosity and surface roughness of textile substrates, however, finally, a surface resistance on polyester fabrics of 0.2 ± 0.025 Ω/sq. in the case of the woven one, and 0.9 ± 0.02 Ω/sq. in the case of the knitted one, has been achieved. The use of graphene in conductive paths was proposed by Afroj et al. [19]. The authors tested a pad-dry-cure method with roller compression and indicated that the obtained resistance was even 11.9 Ω sq^−1^. In addition, resistance to processes simulated utility conditions was provided. Those research papers confirm that textile-based electro-conductive paths is a promising research direction with a great potential of implementation in near future in the fabrics from human surroundings for his/her non-invasive support in everyday life. 

Therefore, in this study authors made attempts to obtain electro-conductive paths on textile fabrics that are more mechanically resistant and resistant to simulated aging cycles. In the publication, the way how to obtain the electrically-conductive material and the results of their laboratory tests are described. The developed paths can contribute to improving the functional characteristics of smart clothing by replacing the rigid electrical connections (between sensors, measuring and control devices and transmission system) with the developed flexible textile material with electro-conductive paths.

## 2. Materials and Methods

### 2.1. Test Materials

#### 2.1.1. Components

Several components were used to prepare test materials that included: polymer, fillers, ionic liquids and textile materials:

- Polymer: ethylene-octene copolymer (EOR)—without a team crosslinking agent, trade name: ENGAGE 8150, the degree of crystallinity: 27%, viscosity acc. to Mooney’s scale (ML(1 + 4) 1210C): 16, producer: The Dow Chemical Company (Midland, MI, USA);
- Electroconductive fillers: (1) electroconductive soot/carbon black (PRINTEX) with a particle size: (10–210) nm (symbol used: PRINTEX); (2) Silver flakes (Silver flakes AX20L) with a particle size: 2.5 µm in average (symbol used: Ag);
- Ionic liquids—manufacturer: Sigma Aldrich (St. Louis, MO, USA): (1) hydrophobic-hexafluorophosphate, 1-hexyl-3-methylimidazolium [hmim][PF_6_], (symbol used: [hmim][PF_6_]); (2) hydrophilic-hexafluorophosphate, 1-butyl-3-methylimidazolium [bmim][PF_6_], (symbol used: [bmim][PF_6_]); (3) hydrophilic-chloride, 1-butyl-3-methylimidazolium [bmim][Cl] (symbol used: [bmim][Cl]).
- Textile materials: three variants of the substrates specified in Table 1 were considered, where variants A and B relate to the same laminate but are differently oriented during the vulcanization process (see Section 2.1.3).

#### 2.1.2. The Compositions of Prepared Polymer Films for Vulcanization with Textile Materials

In order to implement polymeric films for vulcanization with textile materials the following two series of polymer blends differing in the type of fillers were developed:

- in the first series, the polymers containing electrically-conductive carbon black,
- in the second series of polymers with the addition of the silver particles.

The 20 variants of mixtures of carbon black and eight mixtures with the addition of silver were developed. Within each of the groups, different composition mixtures were used: i.e., different filler content, additives in the form of ionic liquids. Alternatively, the polymer extrusion method was used. The compositions of mixtures with carbon black (No. P1–P20) and polymer blends with silver flakes (No. A1–A8) are presented in Table 2.

#### 2.1.3. Technology of Preparation of Electroconductive Polymers, Polymer Films and Vulcanizates

Preparation test materials included a three-stage technological process for which patent no. 232097 was granted by The Patent Office of the Republic of Poland [21]. The samples for the laboratory tests were prepared in the Institute of Technology of Polymers and Dyes, Lodz University of Technology. Firstly, polymer blends were prepared using a laboratory mixer, Brabender GmbH and Co. KG (Duisburg, Germany). The pure polymer was plasticized during approx. 5 min, then the appropriate amount of filler was added and stirring was continued for a further 5 min. The last step was the addition of ionic liquid, where, after application, stirring continued for a further 5 min to homogenize the mixture. The parameters of the mixture:

- the volume of the chamber: V = 80 cm^3^;
- the measuring range torque: 0–200 Nm;
- the ratio of rotating impellers: 2:3;
- the heating medium: paraffin oil;
- the sample mass: 70–80 g;
- the temperature: 60 °C;
- the mixing time: 10 min.

Time of mixture preparation—15 min.

In the second stage, the polymer blends prepared in stage 1 were unified and formed into sheets of thickness of approx. 3 mm, by means of a laboratory mill. The parameters of laboratory mill:

- the length of the cylinders: L = 450 mm;
- the diameter of the cylinders: D = 200 mm;
- the width of the gap between the cylinders: 1.5–3.0 mm;
- the rotational speed of the front roller: Vp = 16 rot./min;
- the friction: 1.0–1.2;
- the average temperature of the cylinders: 30 °C;

Time of mixture preparation—15 min.

In the third stage, the selected polymer mixtures after the rolling process, was extruded on a laboratory microextruder Lab-Station Plasti-Corder N50 Brabender GmbH and Co. KG. The extrusion process consisted of plasticization of the mixture with the screw elements, and next the blend was directed to the extrusion head, forming under pressure the desired shape of the extrudate. A characteristic feature of this process was the possibility of obtaining residue oriented structure. From the rolled/milled mixtures, thin strips were cut out. The supply of the extruder with raw material was carried out as evenly as possible.

After completion of the extrusion of one mixture, in the feed opening pure polymer was placed, and then proceeded to the extrusion of the next mixture. The head of a rectangular mouthpiece was used. During extrusion of mixtures, the speed was controlled in the range (25 to 45) rpm so that the pressure and the temperature inside the head did not exceed the allowable values. The extrudes were made in accordance with ASTM D 2233 [22]. Parameters of the extruder:

- the screw diameter: 19 mm;
- the screw length: 190 mm;
- the ratio of screw length to its diameter (L/D): 10;
- the maximum torque: 150 Nm;
- the maximum operating temperature: 300 °C;
- the maximum pressure by weight: 700 bar;
- the production capacity (0.5–5) kg/h.

Finally, a composite polymer–fabric was prepared. It was made in a rectangular form affixed between the electrically heated shelves of a hydraulic press type E PW-1 with heating panels of overall dimensions approx. 200 × 150 mm, clearance between the heating plates, approx. 100 mm, number of ironing shelves—1. Conditions of the process:

- the temperature of the heating plates: 160 °C;
- the pressure between the plates (in the form)—(100–130) Bar;
- the time in the form: 30 min.

### 2.2. Test Methodology

#### 2.2.1. Surface Resistance Tests 

**Figure 1:**
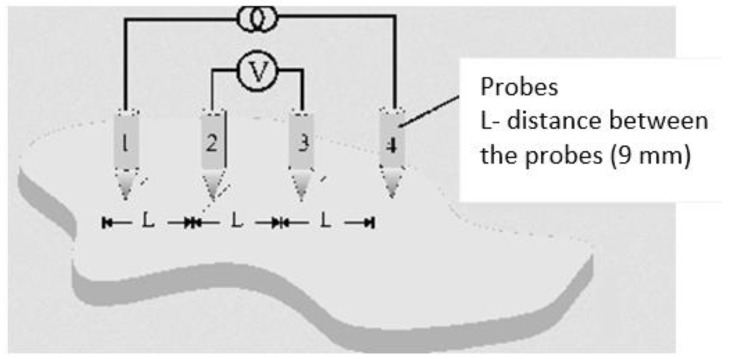
Schematic layout of four electrodes for measurements of surface resistance of electrically-conductive paths by four points method.

For each sample, the surface resistance of the material was measured by means of the four-point method. In the measurements, four electrodes disposed on the same axis at equal distances from one another were used. The scheme of electrode placement is shown in Figure 1.

**Figure 2:**
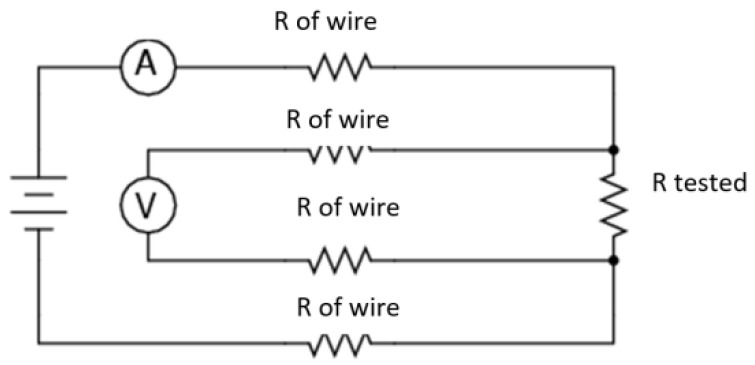
The equivalent circuit of electrical connections in the method used for measuring the surface resistance of textiles.

Due to the different widths of the electrically-conductive materials, measurements were made for each material on 4 mm wide strips, and then the appropriate conversion to surface resistance was applied. For each sample, five measurements were performed. Then, the results for each sample were averaged, and the result was taken as the final value for the sample. In Figure 2, the equivalent circuit of electrical connections in the method used for measuring the surface resistance of electro tracks is shown.

**Figure 3:**
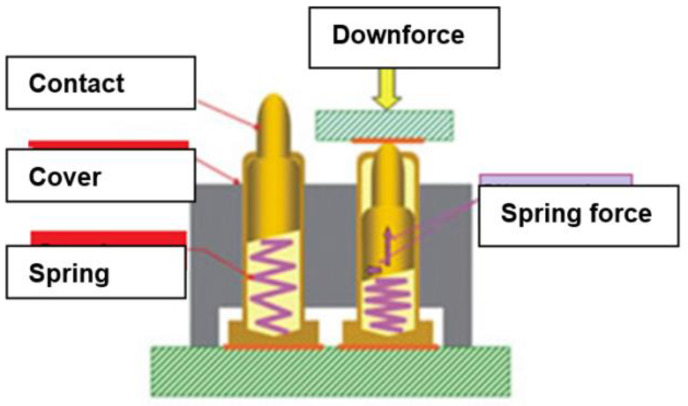
Schematic structure of the electrodes to measure the resistance of textiles.

By means of two outer electrodes, the current was provided while the two inner ones were used for voltage measurement. This eliminates the impact of cable resistance between the voltage meter and measuring points. Each of the electrodes was immersed in the cylindrical plastic and contained in its structure a spring to provide uniform pressure on the substrate. The schematic structure of the electrode is shown in Figure 3.

**Figure 4:**
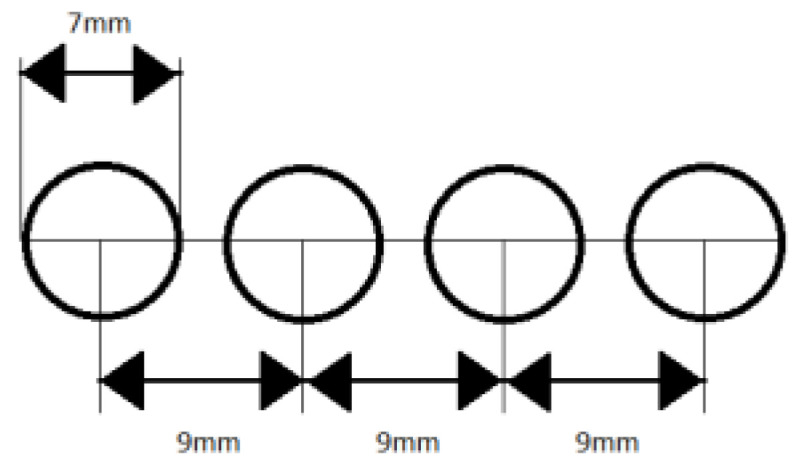
Schematic layout of the holes for electrodes in the acrylic glass.

In order to eliminate the influence of pressure and angle of electrodes to the substrate on the measurement result, the structure stabilizing the position of the electrodes was used. In the acrylic glass panes by means of a laser plotter, four holes with a diameter of 7 mm (diameter matched to the measuring electrodes) at a distance of 9 mm were cut out (Figure 4).

**Figure 5:**
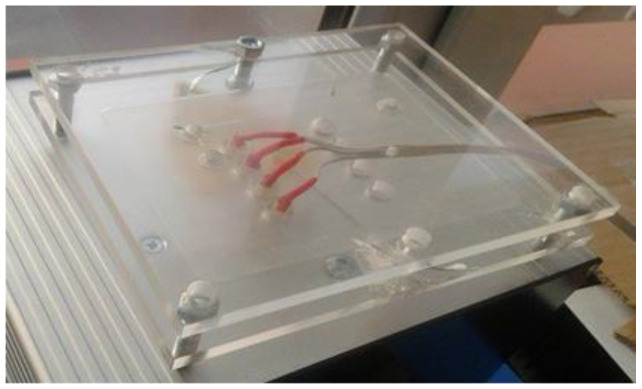
The photo of construction with four electrodes for the measurement of electrical resistance of textiles by the four-point method.

The electrodes to which wires for connection of multimeter had been soldered was placed in the holes. The whole construction was additionally protected by another pane of acrylic glass screwed to the main one. Such a construction enabled placing the burden on it (Figure 5). In measurements, a pressure induced by 1.25 kg of weight was used. The measurements were carried out using two Keithley multimeter sourcemeters 2420 in the atmosphere, for the following parameters: temperature 25 °C, relative humidity 45%.

In order to assess how the simulated aging cycles affect the properties of the electrically-conductive paths developed electro samples were subjected to 10 washing cycles. Washing was performed in accordance with EN ISO 6330:2012 [23]. Washing was performed by hand at 30 °C using a detergent containing no phosphate, no optical brightener and without enzymes.

In the next step, the measurements of surface resistance of electrically-conductive paths after simulated aging cycles that correspond to real utility in the clothing structures were performed and the relative change in resistance was determined according to Equation (1).

(1) $$\Delta R=\frac{R_{0}-R_{k}}{R_{0}}\times 100\%$$

where:

*R*_0_—resistance before simulated aging cycles,

*R_k_*—resistance after simulated aging cycles.

The test materials were also subjected to a series of four bending cycles with the following number of cycles: 10, 20, 30, 50. Bending performed according to ISO 7854:1995 [24] method C. After each series the surface resistance was measured and the relative change in resistance was determined according to Equation (1).

In order to evaluate the mechanical strength of the electrically-conductive materials, their resistance to abrasion was also tested. Abrasion is a form of simulation of the process of garments use as they are vulnerable to friction with the user’s skin or other layers of clothing. In the study Martindale method according to ISO 12947-2:2016 [25] was applied. The surface resistance was measured after 500 cycles of abrasion by wool material. Then, as described above the relative change in resistance was determined (Equation (1)).

#### 2.2.2. Tensile Resistance of Polymer Film (Vulcanizates)

On the basis of the surface resistance results, 5 samples with carbon black and 5 samples with silver flakes were selected for further tests in order to evaluate their mechanical properties. In addition, a reference sample was used, i.e., pure polymer without a filler.

These tests were conducted for vulcanizates i.e., polymer films (prior to vulcanization, with the textile material). The study was conducted in accordance with standard ISO 37:2005 [26] with a machine Zwick Zmart.Pro 1435 Zwick/Roell AG Materialprüfung. In the studies the following parameters were determined:

- tensile strength TS,
- elongation measurement section at break Eb.

#### 2.2.3. Determination of Tear Strength of Composites

Testing the strength of composites (polymer film vulcanized with textile material) to tear was performed in accordance with ISO 34-1:2015 [27]. The samples were prepared in a ”trouser” shape with the following dimensions: 100 mm × 15 mm. Samples were prepared from sheets having a thickness of 2 mm using a press and die-cutting. Samples were cut from the sheet in two different directions. The speed of the jaw during the test was 50 mm/min. Tests were performed in the laboratory in an atmosphere of fixed parameters: temperature 21 °C and 45% relative humidity.

## 3. Results

This section presents results of surface resistance, as well as mechanical properties such as tensile strength and tear resistance. 

### 3.1. Surface Resistance

Table 3 shows the results of measurements of the surface resistance of the developed polymers for EOR/PRINTEX/Ilq and EOR/Ag/Ilq variants.

In the case of both kinds of composites, i.e., with carbon black and silver flakes, the differences in surface resistance after 5 washing cycles, 50 bending cycles and 500 abrasion cycles were not observed. 

### 3.2. Tensile Resistance

In Table 4 the results of the measurement of the tensile strength (elasticity) of selected polymeric films are presented. The results of measurements of the reference sample without filler (Ident. Cat.), as well as the sample with filler, are included.

### 3.3. Tear Strength

Below in Table 5 the results of measurement of tear strength of the composite polymer with filler material for selected polymer films: EOR polymer containing silver and electrically-conductive carbon black are presented.

## 4. Discussion

On the basis of the performed tests, it can be stated that an increase in carbon black content in the mixture resulted in a decrease of electrical resistance RS. The addition of ionic liquids resulted in reduced resistance of vulcanizates in relation to a reference sample that did not contain liquid but only carbon black. Application of an extrusion process did not result in improvement of conductivity, results of RS are in each case slightly higher than for the reference samples (prepared in a conventional manner).

It is worth noticing that according to the conjectures, the highest carbon black content in the compound resulted in the lowest resistance value. The variants containing 30 phr of carbon black were selected for mechanical tests. For samples with silver two series of mixtures of different silver content—25 phr and 50 phr—were performed. As in the case of carbon black, the effect of filler content on the surface resistance was observed. Resistance values of GOhm (to 25 phr of silver) decreased in some cases to kOhm (up to 50 phr). Generally, for samples with a content of 50 phr of silver one can observe the impact of the extrusion process on the resistance values of vulcanizates, a significant influence of two ionic liquids (1 and 2) on the conduction of vulcanizates, in the case of liquids [hmim][PF_6_] and [bmim][PF_6_] resistance values sometimes showed up to 50 Ω, but this depends on the orientation of the sample in relation to the electrode during the measurement, reflecting the heterogeneity of the filler in the polymer. Moreover, in the case of polymeric films, EOR with the content of carbon black and silver no changes in resistance were observed after 50 cycles of bending, after 500 cycles of abrasion and five cycles of washing.

Studying the test results of the tensile strength (TS) and elongation at break (Eb) of the vulcanizates it can be concluded that, regardless of the type of filler, the composites are of the high tensile strength (TS). The lowest strength values were recorded for the reference sample and for the sample with silver and the values of the TS are approx. 10 MPa. For comparison, a reference sample containing only carbon black at 30 phr has a strength of 14 MPa.

The most important goal of this part of the study was to improve the dispersion of the fillers in composites, by the addition of ionic liquids as dispersing agents and it has been achieved. Both in the case of samples with silver or conductive carbon black, the use of selected ionic liquids has improved the strength properties of the rubber vulcanizates (except from variant A7). As in the case of the use of dispersants, positive conclusions can be drawn regarding the effect of the extrusion process employed for the mechanical properties of vulcanizates.

The addition of ionic liquids caused an improvement in the strength of composites rubber from 10 MPa for the reference sample containing only the silver—to approx. 13 MPa—so an increase of over 30% for samples with the addition of two ionic liquids. A smaller, but noticeable, effect can be observed with the use of ionic liquids with respect to the conductive carbon black.

An exception is the test with the addition of 1-butyl-3-methylimidazolium, where the deterioration of the strength characteristics of almost 50% compared to the reference sample. It was most likely caused by the formation of complex chlorine and silver resulting in a high filler agglomeration. This was evident at the very stage of making the composite. The agglomerates of silver concentrated stress what was resulting in deterioration of their mechanical strength.

When using other ionic liquids, it can be concluded that the additive caused a reduction in high surface free energy of fillers particles, which weakened the natural tendency is to aggregation or even agglomeration. The result of applied modification was a more uniform network of filler in polymer complex which was an ethylene-octene rubber. It resulted in the improvement of the reinforcing effect observed in the form of higher static tensile strength values. In further research, special attention to morphological analyses will be paid in order to confirm these observations. 

Considering the samples subjected to the extrusion process, one should also stress the positive aspect of the applied modification of processing the prepared mixtures. The application of extrusion resulted in directing the chain structure of the filler particles in the polymer medium in the direction of the process. Column structures of filler in extrudes resulted in the improvement of the strength of the of rubber vulcanizates, which was observed as an increase in the value TS from 10 MPa for a reference sample with silver to 12.2 MPa for the extraction, and in the case of a sample with carbon black from 14 MPa to 18.7 MPa.

Analysis of the flexibility of composites, measured based on the value of their maximum elongation (Eb) allows to separate two groups of materials, according to the applied fillers:

- Vulcanizates containing carbon black and additives,
- Vulcanizates containing silver and additives.

Vulcanizates with silver are characterized by a significantly higher flexibility which has been measured, the value of Eb ranges from 500–600%. For samples of rubber containing carbon black, it is observed much greater rigidity, which is reflected in the values of elongation at 300%.

Analysis of the test results of composites tear strength of ethylene octane copolymer with textile materials enables to notice a clear division into two groups of materials:

- with improved high tear strength composites with carbon black and the ionic liquid
- high tear strength composites with silver and ionic liquids.

It should be also noted that, regardless of the type of filler or dispersing agents, the strength of material composite has a high value. Characteristics of each of the raw materials used show that already the textile materials—especially fabrics have a high tear strength. There was no apparent effect used the extrusion process on the strength of materials. For the analysis of fabrics A and B, the addition of silver had not significantly improved strength characteristics, in contrast to carbon black, which increased the tear strength Fmit by almost 100%. For material C, both the addition of silver and carbon black has contributed to a significant increase in strength characteristics. In this case, one cannot clearly indicate an upward or downward trend of used additives.

## 5. Conclusions

In this paper a flexible polymer film with two variants of filler: carbon black and silver flakes was subjected to consideration, including various concentrations, as well as ionic liquids applied. On the basis of the performed research, it has been proved that vulcanization technology allows us to obtain electro-conductive textile materials that are characterized by electrical and utility properties adequate for smart clothing applications. In particular, the following conclusions have been formulated taking into account the obtained results:

- The inversely proportional effect of the filler content on the resistance surface was observed. The resistance values in GigaOhms (for 25 phr of silver) have decreased to kiloOhms (for 50 phr of silver).
- For polymer films with 50 phr of silver, there was observed a positive effect of the extrusion process and the addition of ionic liquids on the resistance values of the vulcanizates. In the case of using ionic liquid [bmim][PF_6_], the resistance value even showed 50 kΩ.
- Performed simulated aging cycles did not affect the electrical properties of the polymer films.
- Based on the results of tensile strength tests, it can be concluded that as a result of adding ionic liquids to the polymer as dispersants, the goal of improving the degree of dispersion of fillers in the polymer films was achieved.
- All composites polymer film made of ethylene-octene copolymer fabric showed good mechanical properties in terms of resistance to tearing.
- Further research works are required in order to ensure a higher level of homogeneity of the filler in polymer, as well as to verify the electrical properties under predicted ambient conditions (air humidity in particular).

## 6. Patents

The technology of manufacturing of textile electro-conductive materials presented in the paper is patented (no. 232097, granted by The Patent Office of the Republic of Poland).

## Figures and Tables

**Table 1:** Characteristics of textile materials used in studies.

Symbol	Samples Characteristics	Thickness and Surface Mass
A	Aramid fabric laminated with PES membrane–from the woven fabric side, threads in weft: 224, threads in warp: 324, twill weave	(0.38 ± 0.01) mm (220 ± 22) g/m^2^
B	Aramid fabric laminated with PES membrane–from the membrane side, threads in weft: 224, threads in warp: 324, twill weave	(0.38 ± 0.01) mm (220 ± 22) g/m^2^
C	Cotton fabric coated with polyurethane–film, threads in weft: 460, threads in warp: 670	(0.27 ± 0.01) mm (148 ± 10) g/m^2^

**Table 2:** The compositions of the polymer blends of ethylene-octene copolymer (EOR) with electroconductive carbon black (PRINTEX) and silver flakes (Ag).

No.	EOR	Electroconductive Filler [phr]		Ionic Liquid/Extrusiom
		PRINTEX	Ag	
P1	100	15	-	-/-
P2	100	15	-	[hmim][PF_6_]/-
P3	100	15	-	[bmim][PF_6_]/-
P4	100	15	-	[bmim][Cl]/-
P5	100	15	-	-/extrusion
P6	100	20	-	-/-
P7	100	20	-	[hmim][PF_6_]/-
P8	100	20	-	[bmim][PF_6_]/-
P9	100	20	-	[bmim][Cl]/-
P10	100	20	-	-/extrusion
P11	100	25	-	-/-
P12	100	25	-	[hmim][PF_6_]/-
P13	100	25	-	[bmim][PF_6_]/-
P14	100	25	-	[bmim][Cl]/-
P15	100	25	-	-/extrusion
P16	100	30	-	-/-
P17	100	30	-	[hmim][PF_6_]/-
P18	100	30	-	[bmim][PF_6_]/-
P19	100	30	-	[bmim][Cl]/-
P20	100	30	-	-/extrusion
A1	100	-	25	[hmim][PF_6_]/-
A2	100	-	25	[bmim][PF_6_]/-
A3	100	-	25	[bmim][Cl]/-
A4	100	-	50	-/-
A5	100	-	50	[hmim][PF_6_]/-
A6	100	-	50	[bmim][PF_6_]/-
A7	100	-	50	[bmim][Cl]/-
A8	100	-	50	-/extrusion

**Table 3:** The results of measurements of the surface resistance of EOR/PRINTEX/Ilq and EOR/Ag/Ilq.

No	Composition of EOR Mixture	Surface Resistance
	**PRINTEX/Ilq**	
P1	15	4.5 ± 0.1 kΩ
P2	15/[hmim][PF_6_]	2.5 ± 0.1 kΩ
P3	15/[bmim][PF_6_]	6.4 ± 0.1 kΩ
P4	15/[bmim][Cl]	3.4 ± 0.1 kΩ
P5	15/-(extruded)	6.1 ± 0.1 kΩ
P6	20/-	2.3 ± 0.1 kΩ
P7	20/[hmim][PF_6_]	1.5 ± 0.1 kΩ
P8	20/[bmim][PF_6_]	2.0 ± 0.1 kΩ
P9	20/[bmim][Cl]	1.2 ± 0.1 kΩ
P10	20/-(extruded)	3.1 ± 0.1 kΩ
P11	25/-	1.7 ± 0.1 kΩ
P12	25/[hmim][PF_6_]	1.2 ± 0.1 kΩ
P13	25/[bmim][PF_6_]	1.3 ± 0.1 kΩ
P14	25/[bmim][Cl]	1.0 ± 0.1 kΩ
P15	25/-(extruded)	2.8 ± 0.1 kΩ
P16	30/-	1.5 ± 0.1 kΩ
P17	30/[hmim][PF_6_]	692 ± 20 Ω
P18	30/[bmim][PF_6_]	1.2 ± 0.1 kΩ
P19	30/[bmim][Cl]	962 ± 25 Ω
P20	30/-(extruded)	1.7 ± 0.1 kΩ
	**Ag/Ilq**	
A1	25/[hmim][PF_6_]	23 ± 1 GΩ
A2	25/[bmim][PF_6_]	26 ± 1 GΩ
A3	25/[bmim][Cl]	24 ± 1 GΩ
A4	50/-	1.2 ± 0.1 MΩ
A5	50/[hmim][PF_6_]	1.4 ± 0.1 kΩ
A6	50/[bmim][PF_6_]	50 ± 2 kΩ
A7	50/[bmim][Cl]	19 ± 1 GΩ
A8	50/-(extruded)	110 ± 3 kΩ

**Table 4:** The results of measurements of the tensile strength (TS) and elongation at break (Eb) of developed polymer films.

No	Composite	Tensile Strength [MPa]	Elongation at Break [%]
EOR	EOR ref-pure polymer	10.30 ± 1.21	608 ± 57
	**EOR/Ag/Ilq**		
A4	50	9.97 ± 2.21	606 ± 32
A5	50/[hmim][PF_6_]	13.60 ± 0.99	610 ± 40
A6	50/[bmim][PF_6_]	13.20 ± 0.25	584 ± 36
A7	50/[bmim][Cl]	5.70 ± 0.97	355 ± 44
A8	50 extrusion	12.20 ± 0.71	535 ± 14
	**EOR/PRINTEX/Ilq**		
P16	30	14.00 ± 1.06	304 ± 42
P17	30/[hmim][PF_6_]	14.80 ± 0.43	331 ± 18
P18	30/[bmim][PF_6_]	14.00 ± 0.81	330 ± 28
P19	30/[bmim][Cl]	14.60 ± 0.38	325 ± 18
P20	30 extrusion	18.70 ± 0.84	461 ± 20

**Table 5:** The test results of measurements of tear strength of the composite polymer textile.

No	Composite	Tear Strength [N/mm]		
		A	B	C
	Only textile material	34.8 ± 0.3	34.8 ± 0.2	27.1 ± 2.5
	EOR ref-pure polymer	22.9 ± 1.0	28.7 ± 4.3	44.6 ± 3.3
	**EOR/Ag/Ilq**			
A4	50	33.3 ± 0.13	32.2 ± 0.1	20.4 ± 1.4
A5	50/[hmim][PF_6_]	24.4 ± 1.5	30.6 ± 0.9	34.6 ± 1.0
A6	50/[bmim][PF_6_]	19.5 ± 0.8	29.9 ± 0.7	32.2 ± 2.3
A7	50/[bmim][Cl]	22.7 ± 3.5	26.7 ± 3.6	36.4 ± 1.2
A8	EOR extrusion	21.4 ± 0.5	33.2 ± 4.8	31.0 ± 2.4
	**EOR/PRINTEX/Ilq**			
P16	30	70.6 ± 6.5	64.5 ± 10.1	45.3 ± 5.0
P17	30/[hmim][PF_6_]	53.7 ± 7.5	62.0 ± 9.9	44.6 ± 4.7
P18	30/[bmim][PF_6_]	63.8 ± 1.5	54.3 ± 2.0	42.6 ± 10.6
P19	30/[bmim][Cl]	65.7 ± 11.8	61.0 ± 7.3	51.1 ± 3.3
P20	30 extrusion	66.0 ± 9.3	57.3 ± 4.8	40.0 ± 3.8

## Data Availability

The data presented in this study are available on request from the corresponding author.
